# In Vitro Efficacy of Ultrasonic Debridement with Adjunctive St. John’s Wort on Multispecies Dental Biofilms

**DOI:** 10.3390/medicina62030563

**Published:** 2026-03-18

**Authors:** Zaharia Cristian, Kis Andreea Mihaela, George Andrei Drăghici, Dragoş Vasile Nica, Stefania Dinu, Olariu Iustin

**Affiliations:** 1 Faculty of Dental Medicine, “Victor Babes” University of Medicine and Pharmacy of Timisoara, 9 Revolutiei 1989 Ave., 300070 Timisoara, Romania; cristian.zaharia@umft.ro; 2 Research Center in Dental Medicine Using Conventional and Alternative Technologies, Department of Prostheses Technology and Dental Materials, Faculty of Dental Medicine, “Victor Babes” University of Medicine and Pharmacy of Timișoara, 9 Revolutiei 1989 Ave., 300070 Timisoara, Romania; nicadragos@gmail.com; 3 Management and Communication in Dental Medicine Department, Department I, Faculty of Dental Medicine, “Victor Babes” University of Medicine and Pharmacy of Timisoara, 9 Revolutiei 1989 Ave., 300070 Timisoara, Romania; kis.andreea@umft.ro; 4 Research Center for Pharmaco-Toxicological Evaluations, Faculty of Pharmacy, “Victor Babes” University of Medicine and Pharmacy Timisoara, Eftimie Murgu Square No. 2, 300041 Timisoara, Romania; 5 Faculty of Pharmacy, “Victor Babeș” University of Medicine and Pharmacy Timisoara, Eftimie Murgu Square No. 2, 300041 Timisoara, Romania; 6 The National Institute of Research—Development for Machines and Installations Designed for Agriculture and Food Industry (INMA), Bulevardul Ion Ionescu de la Brad 6, 077190 București, Romania; 7 Department of Pedodontics, Faculty of Dental Medicine, “Victor Babes” University of Medicine and Pharmacy Timisoara, 9 Revolutiei Bv., 300070 Timisoara, Romania; dinu.stefania@umft.ro; 8 Pediatric Dentistry Research Center, Faculty of Dental Medicine, “Victor Babes” University of Medicine and Pharmacy Timisoara, 9 Revolutiei Bv., 300070 Timisoara, Romania; 9 Department of Dentistry, Faculty of Dentistry, “Vasile Goldiş” Western University of Arad, 94–96 Revoluţiei Blvd., 310025 Arad, Romania; olariu.iustin@uvvg.ro

**Keywords:** *Hypericum*, biofilms, *Porphyromonas gingivalis*, periodontal debridement, dental plaque

## Abstract

*Background and Objectives*: The use of St. John’s wort (*Hypericum perforatum*) in periodontal therapy remains underexplored despite its anti-inflammatory, antimicrobial, and potential osteoregenerative effects. This was the first study aiming to determine the *in vitro* efficacy of ultrasonic debridement combined with a *H. perforatum* extract against dental biofilms. *Materials and Methods*: A multispecies biofilm model comprising *Streptococcus mutans*, *Porphyromonas gingivalis*, *Fusobacterium nucleatum*, and *Tannerella forsythia* was established on bovine dentin discs. Biofilms were treated with saline solution (control), ultrasonic debridement alone, ultrasonic debridement combined with *H. perforatum* extract (0.5%), and ultrasonic debridement combined with chlorhexidine (0.12%). Biofilm biomass was quantified with the crystal violet assay, and total viable counts were determined by colony-forming unit (CFU) analysis. Quantitative PCR was used to assess the genomic load of *P. gingivalis*. Biofilm architecture and bacterial viability were further examined using confocal laser scanning microscopy (CLSM). *Results:* Ultrasonic debridement combined with *H. perforatum* extract significantly reduced biofilm biomass compared to saline irrigation (*p* < 0.001) and ultrasonic debridement alone (*p* < 0.01). Similar reductions were observed for viable bacterial counts and *P. gingivalis* genomic load. The antimicrobial effect of the plant extract was comparable to that of chlorhexidine, with only minor differences in efficacy. Confocal microscopy confirmed marked disruption of biofilm architecture and decreased bacterial viability following treatment with the plant extract. *Conclusions*: Within the limitations of this *in vitro* model, *H. perforatum* extract demonstrated measurable antibiofilm activity when used as an adjunct to ultrasonic debridement. These findings provide proof-of-concept evidence supporting the antimicrobial potential of this plant-derived extract under controlled laboratory conditions. Further preclinical studies and well-designed clinical investigations are required to determine its therapeutic relevance in periodontal treatment.

## 1. Introduction

The oral microbiota play a key role in human health [1]. Under normal conditions, biofilms formed and maintained by these bacterial consortia on surfaces inside the mouth act as a frontline defense against pathogenic species, and a potent modulator of immune maturation and tolerance [2,3]. Dysbiosis of the oral microbiota creates an inflammatory environment characterized by a shift from commensal Gram-positive species to pathogenic anaerobic bacteria [4]. This disruption of host–microbe interactions promotes persistent low-grade inflammation and ineffective pathogen clearance, ultimately contributing to tissue destruction and periodontal diseases [5]. The progression from gingivitis to periodontitis is not linear, but occurs in episodic bursts of activity, moving from gingival inflammation to loss of connective tissue attachment, periodontal pocketing, alveolar bone resorption, and tooth mobility and loss [6]. Mechanical plaque removal by scaling and root planing (SRP) is typically the first-line treatment for periodontitis, with adjunctive use of antiseptics for local bacterial control and/or antibiotics for severe or refractory cases [7,8].

Bacterial biofilms undergo maturation and transformation onto tooth surfaces, transitioning from biofilm (microscopic level) to dental plaque (clinically visible biofilm) and dental calculus (mineralized biofilm) [9,10]. Ultrasonic debridement is widely used to disrupt and remove biofilm deposits along this continuum. This procedure can remove bacterial biofilm and flush debris from periodontal pockets up to 8–9 mm [11,12], and therefore, it is considered equally effective as non-surgical periodontal therapy (manual scaling) for treating periodontitis [13]. Complete eradication of biofilms in complex root anatomies and deep periodontal pockets, however, remains challenging, with residual bacterial loads acting as potential drivers of disease recurrence [14,15]. In this context, adjunctive antimicrobial therapies have attracted increasing research interest as strategies to enhance the effectiveness of mechanical debridement.

With a history of use in folk and traditional medicine dating back to the Ancient Greeks, St. John’s wort (*Hypericum perforatum* L.) exerts a wide variety of pharmacological effects, including anti-inflammatory, antimicrobial, and wound-healing action [16]. Hypericin and hyperforin are the major active constituents underlying these effects: hyperforin is a potent bactericidal agent (especially against Gram-positive bacteria) [17], while hypericin can be activated via light to remove biofilms and regulate inflammation [18]. Several *in vitro* studies have demonstrated the antibacterial and anti-biofilm effects of *Hypericum* extracts against oral periodontopathogens [19,20,21]. *In vivo* investigations conducted on rodent models (mainly rats) confirmed these results. It was demonstrated that St. John’s wort preparations reduce inflammation, alveolar bone loss, and oxidative stress while limiting collagen degradation and improving regenerative outcomes of xenograft-filled bone defects [22,23,24,25,26,27,28]. Moreover, *Hypericum*-based products have demonstrated comparable outcomes with chlorhexidine in two early clinical trials, with broadly similar effects in reducing dental plaque, gingival inflammation, and complications after third molar removal [29,30]. However, its direct application as an antimicrobial irrigant during mechanical debridement for dental biofilm management remains underexplored. Furthermore, most studies have evaluated its effects in planktonic cultures, mono-species biofilms, or animal models, although the antimicrobial properties of *H. perforatum* have been previously described in oral microbiology [16,18,21,24,29,30].

In this context, we aimed to extend existing knowledge by investigating the effect of a hydroalcoholic *H. perforatum* extract within a mechanically disrupted multispecies dentin-disc biofilm model, simulating its potential use as an adjunctive irrigant during ultrasonic periodontal debridement. Our hypothesis was that the combined use of ultrasonic debridement and irrigation using an *H. perforatum* preparation is superior to ultrasonic debridement with saline in decreasing biofilm biomass and bacterial viability. Chlorhexidine was used as a reference compound since it is considered the gold standard antimicrobial agent for plaque control and adjunctive periodontal therapy [31]. Multiple complementary outcome measures—including biofilm biomass, viable bacterial counts, qPCR quantification of *Porphyromonas gingivalis*, and confocal microscopy—were used to provide a multidimensional evaluation of antibiofilm activity. These findings may be relevant for researchers investigating biofilm control strategies and for the development of plant-derived adjuncts to conventional periodontal therapy.

## 2. Materials and Methods

This study was designed as a controlled *in vitro* experimental investigation evaluating the antimicrobial effects of different irrigation protocols during ultrasonic debridement on a multispecies dentin-disc biofilm model. Each group included ten dentin discs (*n* = 10). A formal *a priori* power analysis was not performed, as the study was conceived as an exploratory proof-of-concept experiment. The selected sample size is consistent with commonly used designs in biofilm studies and was considered sufficient to detect treatment-related differences under controlled laboratory conditions. As no clinical examiners or operator-dependent measurements were involved, examiner calibration procedures were not required. All experimental procedures were conducted under standardized laboratory conditions according to predefined protocols to ensure reproducibility.

### 2.1. Biofilm Model

Sterilized standardized bovine dentin discs (7 mm diameter and 1 mm thickness; Modus Laboratories, Reading, UK) were used as substrates for biofilm growth. The multispecies biofilm model included the early colonizer *Streptococcus mutans* (ATCC 25175) and key periodontal pathogens *Porphyromonas gingivalis* (ATCC 33277), *Fusobacterium nucleatum* (ATCC 25586), and *Tannerella forsythia* (ATCC 43037). *S. mutans* was included as an early colonizing species capable of initiating biofilm formation on dentin surfaces and facilitating the subsequent establishment of anaerobic periodontal pathogens [32]. *P. gingivalis*, *F. nucleatum*, and *T. forsythia* represent clinically relevant periodontal pathogens frequently associated with dysbiotic subgingival biofilms.

*S. mutans* was cultivated on Brain Heart Infusion (BHI) agar (BD Difco, Franklin Lakes, NJ, USA) at 37 °C in a bacteriological incubator at a partial pressure of 5% CO_2_ [33]. *P. gingivalis* was grown on BHI agar supplemented with 5 µg/mL hemin (Merck KGaA, Darmstadt, Germany) and 1 µg/mL vitamin K_1_. *F. nucleatum* was cultivated on Brucella agar (Oxoid Ltd., Basingstoke, UK) supplemented with the same additives. *T. forsythia* was cultured on tryptic soy agar (Merck KGaA, Darmstadt, Germany) supplemented with 5% defibrinated sheep blood, 5 µg/mL hemin, 1 µg/mL vitamin K_1_, and 10 µg/mL N-acetylmuramic acid (Merck KGaA, Darmstadt, Germany).

Bacteria were cultured in appropriate media and then mixed in equal volumes to achieve a final optical density (OD600) of 0.5. Biofilms were grown in 24-well plates under anaerobic conditions (80–85% N_2_, 5–10% CO_2_, 10% H_2_) in an anaerobic chamber (Whitley DG250, Don Whitley Scientific, Bingley, UK) at 37 °C for 7 days. The culture medium was replenished daily to allow biofilm maturation.

### 2.2. Experimental Procedures

A standardized hydroalcoholic extract of *Hypericum perforatum* containing 0.3% hypericin and 3% hyperforin was obtained from a reputable local supplier (Dacia Plant, Bod, Romania). The extract was obtained using an ethanol–water (approximately 70% ethanol *v*/*v*) hydroalcoholic solvent system. All experiments were conducted with the same batch of the extract to minimize batch-to-batch variability in phytochemical composition. The stock preparation was stored protected from light at 4 °C to preserve the stability of photosensitive constituents such as hypericin, and working solutions were freshly prepared before each experimental run.

A 0.5% (*w*/*v*) extract solution prepared in sterile 0.9% NaCl was used as a control, chlorhexidine digluconate (0.12% CHX) as a positive antimicrobial control, and sterile physiological saline (0.9% NaCl) as a negative control. To date, there is no established standardized antimicrobial concentration range for hydroalcoholic *Hypericum perforatum* extracts in dentin-disc biofilm models. We therefore selected a practical working concentration for exploratory evaluation in the present proof-of-concept study, allowing exposure of the biofilm to the extract’s bioactive constituents—including hypericin, hyperforin, and phenolic compounds. The selected concentration (0.5% *w*/*v*) aligns with the ‘low-interaction’ safety profile recognized by the European Medicines Agency (EMA) and ensures the final solvent levels remain below the threshold required to avoid confounding cytotoxicity in biological assays [34,35]. This concentration also falls within ranges commonly used in experimental phytochemical and antimicrobial investigations of *Hypericum* extracts, where standardized preparations containing hypericin and hyperforin are evaluated for biological activity.

Mature biofilm-laden dentin discs were randomly allocated to each experimental groups and processed as follows: (*i*) for the untreated control (UC), discs were left undisturbed; (*ii*) for saline control (SC), discs were irrigated with sterile saline solution; (*iii*) ultrasonic debridement alone (UD), discs underwent ultrasonic debridement with continuous irrigation using sterile saline solution; (*iv*) ultrasonic debridement with St. John’s wort (UD + SJW), discs underwent ultrasonic debridement with continuous irrigation using the 0.5% SJW extract solution; (*v*) ultrasonic debridement with chlorhexidine (UD + CHX), discs underwent ultrasonic debridement with continuous irrigation using 0.12% chlorhexidine. All treatments (except controls) lasted 30 s.

For ultrasonic debridement, we used an EMS Piezon Master 700^®^ (EMS Dental, Nyon, Switzerland). This laboratory-grade piezoelectric scaler operates at a vibration frequency of approximately 30 kHz within a 24–32 kHz range, with a maximum output power capacity of ~12 W under load. The universal periodontal PS tip was mounted according to the manufacturer’s instructions, and the device power was set to 30% of the maximum output, corresponding to a moderate clinical setting for periodontal debridement [36]. The tip was applied with gentle pressure following a standardized sweeping motion across the biofilm surface for 30 s per disc, with the irrigation being delivered at a constant flow rate (20 mL/min) throughout the debridement process. This timeframe was selected based on *in vitro* protocols for ultrasonic instrumentation. Literature reports indicate that application times of 30–60 s are commonly used for periodontal ultrasonic scaler tips in biofilm removal studies, as this duration allows effective debris and biofilm removal while preserving dentin integrity [37,38].

### 2.3. Outcome Measures

We used the crystal violet assay to quantify biofilm biomass. The discs with biofilm were stained with 0.1% solution of crystal violet (Merck KGaA, Darmstadt, Germany) for 15 min at room temperature. The bound dye was solubilized post-rinse with 33% acetic acid, and its absorbance at 595 nm was measured with a Multiskan FC microplate photometer (Thermo Fisher Scientific, Waltham, MA, USA).

To assess total viable bacterial count, biofilms were detached from the dentin discs through vortexing and sonication in sterile saline. Serial (tenfold) dilutions were prepared and plated onto appropriate agar media (see above). After incubating the plates under anaerobic conditions for 48–72 h, visible colonies were counted to determine colony-forming units per disc (CFU/disc). The results obtained were expressed as the logarithm (base 10) of colony-forming units per disc (Log_10_ CFU/disc).

Bacterial DNA was recovered from biofilms using the ZR Fungal/Bacterial DNA Miniprep™ Kit (Zymo Research, Inc., Irvine, CA, USA) as per the manufacturer’s instructions. Real-time qPCR analysis was conducted to determine the quantity of DNA of *Porphyromonas gingivalis* in samples using the following primers and probes: forward primer (20 nt): 5′- AGGCAGCTTGCCATACTGCG-3′; reverse primer (21 nt): 5′- ACTGTTAGCAACTACCGATGT-3′; probe (30 nt, FAM/TAMRA): 5′-FAM GCTAATGGGACGCATGCCTATCTTACAGCT-TAMRA-3′ [31]. We focused exclusively on this Gram-negative anaerobic bacterium given its role as a key periodontal pathogen, whose presence and relative abundance are closely linked to oral dysbiosis and the progression of periodontal disease [30]. Quantification of *P. gingivalis* genomic load, therefore, serves as a surrogate indicator of changes in the pathogenic component of the biofilm, allowing targeted evaluation of treatment effects on a clinically relevant organism. Each 20 µL reaction mix contained 10 µL TaqMan Universal Master Mix (Applied Biosystems, Foster City, CA, USA), 400 nM of each primer, 200 nM probe, 2 µL of DNA template, and nuclease-free water. Amplification was performed in an Applied Biosystems (ABI) 7500 FAST Real-Time PCR system (Applied Biosystems, Foster City, CA, USA) under the following cycling conditions: 95 °C for 2 min, 40 cycles of 95 °C for 15 s, and 60 °C for 1 min, with fluorescence acquisition during the annealing/extension step [31]. A standard curve was created using tenfold serial dilutions of purified *P. gingivalis* genomic DNA (10^1^–10^7^ genome copies per reaction) for absolute quantification. Each run for contamination control included no-template controls and extraction blanks.

Confocal laser scanning microscopy (CLSM) was used for qualitative biofilm visualization. For each treatment group, discs were stained with a LIVE/DEAD ^®^ BacLight ^TM^ Bacterial Viability Kit (BacLight Kit) (Thermo Fisher Scientific, Waltham, MA, USA). This is aimed at separating live (green fluorescence) from dead (red fluorescence) bacteria within the biofilm structure. Images were acquired with a confocal laser scanning microscope (Leica TCS SP8, Leica Microsystems GmbH, Wetzlar, Germany) using a 63× oil immersion objective, resolution of 1024 × 1024 pixels, and a z-step size of 0.5 µm. All analyses were performed in triplicate using independently prepared samples to ensure reproducibility and reliability of the results.

### 2.4. Statistical Analysis

Statistical analyses were performed using the Statistica 8 software package (StatSoft Inc., Tulsa, OK, USA; STATISTICA, version 8.0, http://www.statsoft.com). Data sets were checked for normality and homogeneity of variance using respectively Kolmogorov tests and Levene’s tests [39]. A one-way ANOVA was applied to compare treatment groups when these assumptions were met, with Tukey HSD tests being used for multiple comparisons. When normality assumptions were violated, non-parametric tests were used, i.e., Kruskal–Wallis with Dunn’s post hoc test and Holm–Bonferroni adjustment in case of significant differences [40]. A *p*-value less than 0.05 was considered significant for all statistical analyses.

## 3. Results

All variables showed normal distributions (Kolmogorov tests, *p* ≥ 0.053) and homogeneous variances (Levene’s tests, *p* ≥ 0.062). As a result, one-way ANOVA was the method of choice for comparing the effect of different treatments.

### 3.1. Biofilm Mass Reduction

**Figure 1:**
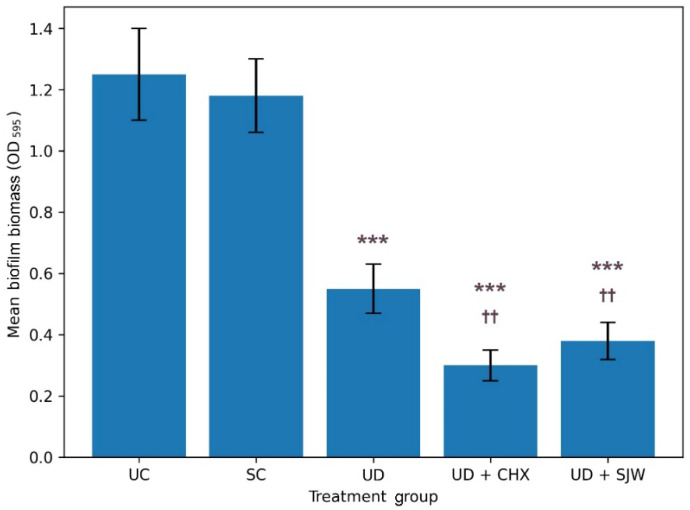
Crystal violet–stained biofilm mass measured as optical density (595 nm) in different treatment groups. The data are given as mean (bar) with one standard deviation (error bar). Bars marked with asterisks (*) indicate statistical differences versus baseline control. Bars marked with dagger symbols (†) denote statistical differences versus ultrasonic debridement alone (Tukey’s HSD test, ***/†††—*p* < 0.001, **/††—*p* < 0.01, */†—*p* < 0.05). UC, untreated control; SC, saline control; UD, ultrasonic debridement alone; CHX, chlorhexidine; SJW, *H. perforatum* extract.

Figure 1 shows the mean biofilm mass for each treatment group. Detailed statistical results are provided in Supplementary Table S1. We observed significant intergroup differences in biofilm amount (ANOVA, *p* < 0.001). Saline irrigation produced biofilm levels comparable to the untreated control, whereas ultrasonic debridement significantly reduced biofilm accumulation. The largest reduction in biofilm biomass was observed for ultrasonic debridement combined with chlorhexidine, followed closely by ultrasonic debridement with *H. perforatum* extract.

### 3.2. Total Viable Bacterial Count Reduction

**Figure 2:**
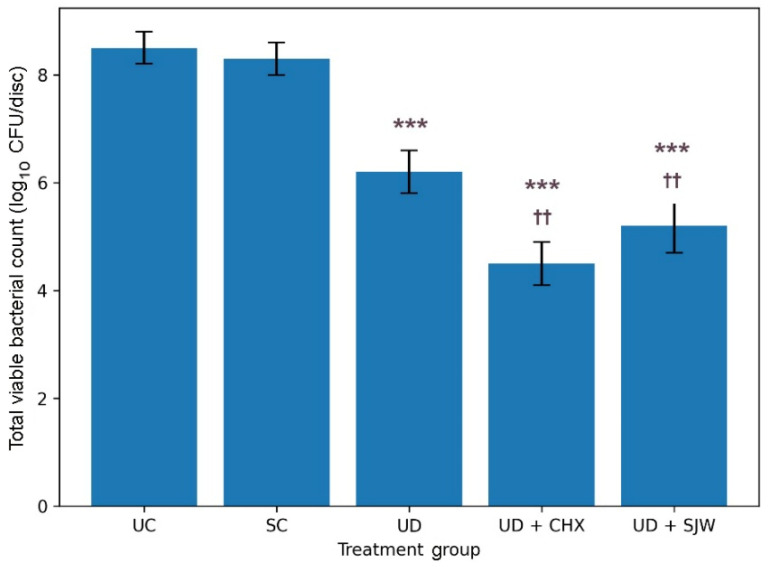
Total viable bacterial count in different treatment groups. The data are given as mean (bar) with one standard deviation (error bar). Bars marked with asterisks (*) indicate statistical differences versus baseline control. Bars marked with dagger symbols (†) denote statistical differences versus ultrasonic debridement alone (Tukey’s HSD test, ***/†††—*p* < 0.001, **/††—*p* < 0.01, */†—*p* < 0.05). UC, untreated control; SC, saline control; UD, ultrasonic debridement alone; CHX, chlorhexidine; SJW, *H. perforatum* extract.

Figure 2 illustrates the total viable bacterial counts for each treatment group. Detailed statistical data are given in the Supplementary Table S2. Although the measured values differed significantly across groups (ANOVA, *p* < 0.001), similar CFU counts were found in normal saline irrigation and controls. Ultrasonic debridement alone yielded a significant decrease in bacterial count compared to the non-treated group. The use of ultrasonic debridement together with St. John’s wort irrigation was associated with an even greater reduction in total CFU counts relative to both untreated and saline controls. Combined ultrasonic debridement and chlorhexidine therapy, on the other hand, resulted in a modestly lower total viable bacterial count compared to ultrasonic debridement in combination with St. John’s wort irrigation.

### 3.3. Treatment-Specific Reduction in Porphyromonas gingivalis

**Figure 3:**
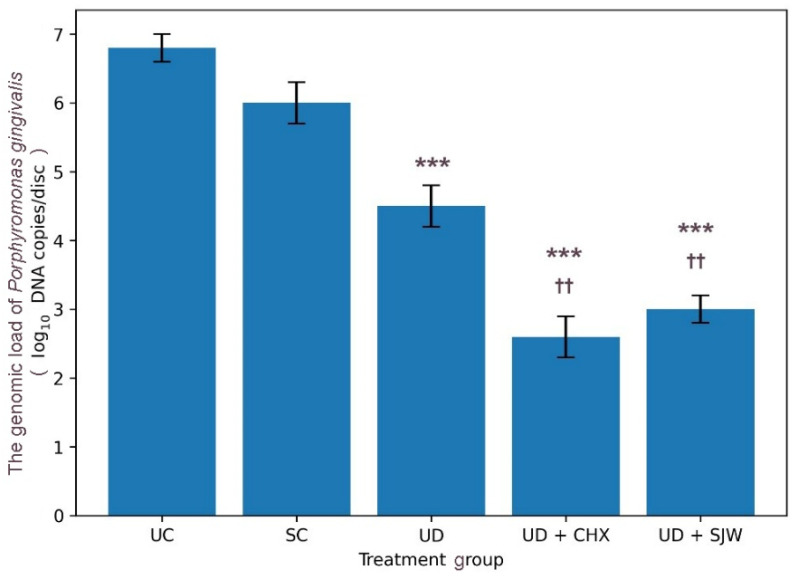
*Porphyromonas gingivalis* genomic load (log10 DNA copies/disc) after treatments. The data are given as mean (bar) with one standard deviation (error bar). Bars marked with asterisks (*) indicate statistical differences versus UC (baseline control). Bars marked with dagger symbols (†) denote statistical differences versus ultrasonic debridement alone (Tukey’s HSD test, ***/†††—*p* < 0.001, **/††—*p* < 0.01, */†—*p* < 0.05). UC, untreated control; SC, saline control; UD, ultrasonic debridement alone; CHX, chlorhexidine; SJW, *H. perforatum* extract.

Figure 3 presents the genomic load of *P. gingivalis* across groups. Detailed statistical results are reported in Supplementary Table S3. This variable demonstrated significant intertreatment variability (ANOVA, *p* < 0.001). There was a marked decline in the relative abundance of *P. gingivalis* in ultrasonic debridement versus untreated control or saline control. Combined ultrasonic debridement and *H. perforatum* extract significantly reduced the genomic load of *P. gingivalis*, decreasing values to less than half of baseline levels. However, ultrasonic debridement in conjunction with chlorhexidine produced only a marginally improved outcome. These differences remained significant when ultrasonic debridement alone was compared to the combined treatment approach.

### 3.4. Qualitative Biofilm Visualization (CLSM)

**Figure 4:**
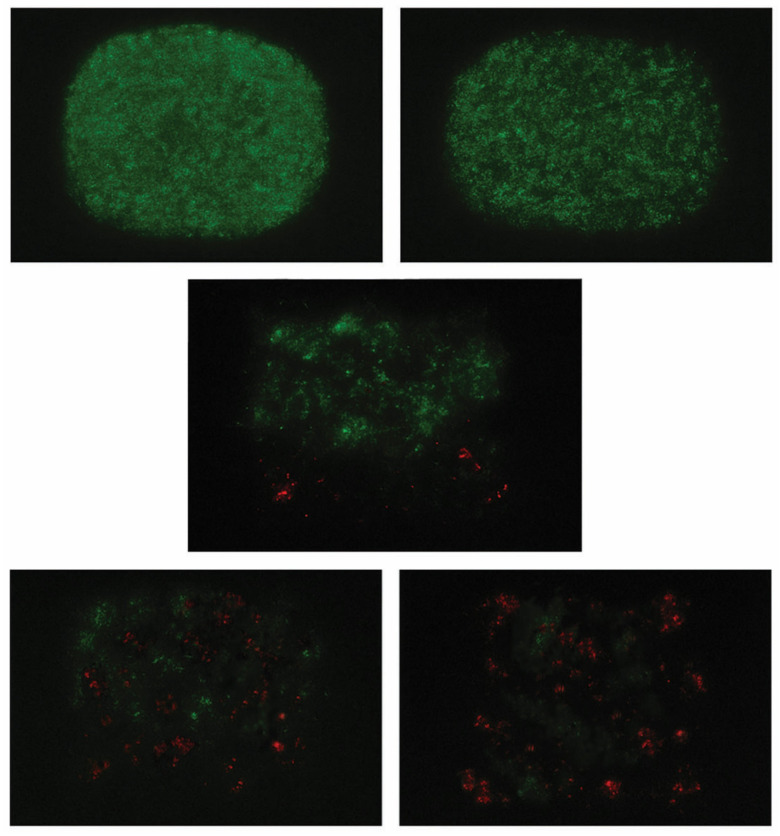
Representative CLSM images of bacterial biofilms from different treatment groups. The control group (**top left corner**) showed densely aggregated, rounded biofilm structures with homogeneous fluorescence intensity. For the saline control (**top right corner**), a similar fluorescence distribution but with a slightly lower packing density was observed. Ultrasonic debridement alone (**middle bottom**) disrupted biofilm organization, yielding patchy green fluorescence interspersed with localized red fluorescence. When combined with St. John’s extract (**bottom left corner**) and chlorhexidine (**bottom right corner**), this treatment induced sparse biofilm, with increased red fluorescence and scattered green.

Representative CLSM images are shown in Figure 4. The pattern observed in untreated controls, with dense, rounded, and uniform green fluorescence, is indicative of high bacterial viability with intact biofilm architecture (Figure 4, top left). A similar fluorescence, but with less compact biofilm, was observed for the saline control group (Figure 4, top right), supporting that this treatment caused only a minor reduction in bacterial viability. In contrast, ultrasonic debridement yielded moderate biofilm alterations, with heterogeneous green fluorescence (viable cells) and scattered red-stained (non-viable) areas (Figure 1, middle center). This approach led to increased red fluorescence and patchy green fluorescence when used in conjunction with St. John’s wort irrigation (Figure 4, bottom left), implying reduced bacterial viability. The appearance of biofilm exposed to combined ultrasonic and chlorhexidine treatment suggests a slightly more potent antibacterial effect compared with the latter treatment (Figure 4, bottom right).

## 4. Discussion

Given the global surge in antibiotic resistance over the past two decades, the general consensus shifted towards a more rational use of these drugs as an adjunct in non-surgical periodontal therapy; for example, in aggressive periodontitis and after antibiotic susceptibility testing [41]. Under these circumstances, the use of medicinal plant extracts against periodontopathic bacteria emerged as a viable alternative. This is the first study investigating the effect of St. John’s wort extracts as an antimicrobial irrigant during mechanical debridement on oral microbiota. Evidence to date has been derived from the use of topical rinses, oils, or systemic administration in animal models [22,23,24,25,29,30]. In addition, none of the previous studies evaluating *Hypericum perforatum* in periodontal or oral contexts employed confocal laser scanning microscopy to assess biofilm viability or architecture. Specifically, antimicrobial and anti-biofilm effects were determined using culture-based methods and optical density measurements [19,20,21], whereas animal studies have focused on histological, biochemical, or histomorphometric outcomes [22,23,24,25]. The novelty of the present study lies not in demonstrating the antimicrobial activity of *H. perforatum* itself—which has been previously reported—but in evaluating its effect in a mechanically disrupted multispecies biofilm model designed to approximate adjunctive irrigation during ultrasonic periodontal debridement. The use of multiple analytical modalities provided a multidimensional evaluation of antibiofilm activity under controlled experimental conditions.

In this study, we used complementary metrics to demonstrate that a hydroalcoholic extract of *H. perforatum* exerts notable antibiofilm activity across three different dimensions: physical (bacterial biomass), biological (viability), and structural (visual architecture). These results are in agreement with an expanding body of (pre)clinical evidence. Thus, Bagheri et al. have investigated the antimicrobial activity of *H. perforatum* oil against *Enterococcus faecalis*, *Escherichia coli*, *Streptococcus mutans*, *Staphylococcus aureus*, and *Porphyromonas gingivalis*. When administered as pure oil or 50% diluted with dimethyl sulfoxide (DMSO) 0.1%, this product yielded diameters of inhibition zones similar to those observed for ciprofloxacin, gentamicin, amikacin, amoxicillin, and metronidazole [19]. These outcomes are in line with our findings, as the application of *H. perforatum* resulted in a marked reduction in biofilm biomass and viable bacterial counts. However, unlike previous *in vitro* studies [19,20,21], we evaluated the effect of *H. perforatum* on a pre-established multispecies biofilm following mechanical disruption—a condition that more closely reflects the clinical periodontal environment.

Arpağ et al. have examined the impact of essential oil extracted from the St. John’s wort flowers on *P. gingivalis* versus povidone-iodine and chlorhexidine given at clinically relevant concentrations, i.e., 10% and 0.2%, respectively. This plant-derived product induced a significant fourfold reduction in minimum inhibitory concentration versus povidone-iodine, and a significant twofold decrease versus chlorhexidine [20]. These findings suggest that *Hypericum*-derived preparations may possess antimicrobial activity comparable to commonly used antiseptics under certain experimental conditions. Indeed, our data provide such evidence when comparing the antimicrobial effectiveness of *H. perforatum* hydroalcoholic extract and chlorhexidine against *P. gingivalis.* These data also support a high susceptibility of *Porphyromonas gingivalis* to *H. perforatum*, which is concordant with our qPCR results.

The limited effect seen here for saline irrigation alone supports that mechanical flushing without antimicrobial activity is insufficient for effective biofilm control [39], reinforcing the necessity of adjunctive antimicrobial strategies. Ultrasonic debridement, by contrast, significantly reduced biofilm biomass and bacterial load. This is in line with the well-established role of mechanical instrumentation in non-surgical periodontal therapy [42]. Previous studies often report higher bacterial reductions after ultrasonic debridement (in some cases even exceeding 90%), but methodological differences hinder their comparison with our results. These studies have typically evaluated biofilm removal from enamel or polished root surfaces, substrates that do not harbor protected bacterial reservoirs due to the absence of dentinal tubules. On the other hand, dentin contains a dense tubular network that permits microbial penetration and persistence beneath the surface, limiting the effectiveness of mechanical instrumentation. Furthermore, sampling methods that recover bacteria from dentinal tubules may yield lower apparent reductions compared with studies assessing only superficial plaque removal. Differences in biofilm maturity and the absence of physiological fluid dynamics in *in vitro* models may further contribute to reduced debridement efficacy [43,44,45].

The additive effect of mechanical biofilm disruption and phytotherapeutic antimicrobial action of St. John’s wort extract observed here is also noteworthy. While this synergy suggests that *H. perforatum* extract enhances mechanical biofilm disruption *in vitro*, its clinical performance as an antimicrobial irrigant during mechanical debridement remains to be fully clarified. This aspect is important, since in a clinical setting, factors such as salivary dilution and the presence of gingival crevicular fluid may influence the retention and activity of the extract differently than in our controlled irrigation protocol. The potential of this approach is, however, underpinned by the modest differences between *H. perforatum* and chlorhexidine, with chlorhexidine producing only a modest increase in antimicrobial activity. Similar results were reported in other studies. For example, Nisha et al. evaluated the effects of St. John’s wort mother tincture on periodontal health in 418 patients. Use of a mouthwash containing this product (1:4 dilution) significantly lowered plaque burden and gingival inflammation in comparison with a saline mouthwash. Chlorhexidine showed a superior effect at three, but not at six-month follow-up [29]. These effects are broadly consistent with our findings.

However, dental literature reports adverse effects associated with prolonged use of chlorhexidine, including altered taste perception (typically a bitter or metallic sensation), brown/yellowish staining on teeth, tongue, and fillings, and cytotoxicity for osteoblasts, fibroblasts, and myoblasts [46]. In contrast, St. John’s wort–based products revealed anti-inflammatory and regenerative effects in both animal and clinical studies. Thus, Tanideh et al. found that a *H. perforatum* hydroalcoholic extract lowers inflammation, oxidative stress, and alveolar bone loss in a rodent model of ligature-induced periodontitis (adult male Sprague–Dawley rats) when given alone or in combination with a hydroalcoholic extract of *Calendula officinalis* [22]. Using the same animal strain and disease model, Paternitti et al. showed that a *Hypericum* methanolic extract significantly reduces alveolar bone loss, neutrophil infiltration, leukocyte recruitment, production of nuclear factor kappa B (NF-κB) and inducible nitric oxide synthase (iNOS), tyrosine nitration, and activation of the nuclear enzyme poly (ADP-ribose) polymerase, while preventing the loss of antipoptotic pathways [13]. On the other hand, Halicioglu et al. observed that a crude extract of *H. perforatum* significantly increases capillary neogenesis, osteoclast numbers, inflammatory cell infiltration, and new bone formation at the premaxillary suture in albino Wistar male rats [24]. Similar effects on bone regeneration were reported by Damlar et al. in calvarial bone of male New Zealand rabbits: a St. John’s wort oil extract improved bone healing in rabbit skull defects filled with bovine xenografts, increasing new bone formation and reducing residual graft volume compared to controls [23]. Based on the antibiofilm efficacy observed in this *in vitro* model, *H. perforatum* represents a promising candidate for further preclinical investigation as a potential adjunctive antimicrobial strategy. Nevertheless, clinical safety profiles—specifically potential staining or soft tissue irritation over repeated applications—must be rigorously evaluated before it can be recommended as a clinical substitute for the current gold standards.

When compared with controls, saline irrigation, or ultrasonic debridement alone, the analysis of qPCR data disclosed a significant decrease in *P. gingivalis* bacterial burden following combined ultrasonic debridement and *H. perforatum* irrigation. This is a key finding since previous investigations on this topic—such as that of Arpağ et al. [20]—were conducted in planktonic (free-floating) cultures, not in biofilms. This reduction in *P. gingivalis* DNA suggests a targeted suppression of this keystone pathogen. On the other hand, it is known that qPCR detects total DNA, including DNA from non-viable cells. Therefore, while these results indicate a significant reduction in the genomic load, the actual impact on the microbial shift within a patient’s subgingival ecosystem requires a long-term longitudinal study.

It is worth noting that we applied CLSM here for direct visualization of biofilm viability in response to St. John’s wort extract application, unlike earlier published research, which relied exclusively on culture-based, histological, or clinical outcome measures [19,20,21,22,23,24,25,26,27,28,29]. Since this procedure was qualitative and intended primarily to provide descriptive visualization of biofilm architecture and bacterial viability patterns post-treatment, these observations should be interpreted as supportive evidence complementing the quantitative findings from biomass measurements, viable bacterial counts, and qPCR analysis, rather than as independent proof of antimicrobial efficacy. This multidimensional approach suggests that combined treatments may induce substantial loss of bacterial viability and disruption of biofilm architecture despite the purely qualitative nature of the microscopic analysis. Indeed, dental literature shows that a 0.12% CHX solution kills a large proportion of bacteria; for example, Pela et al. reported that nearly three-quarters of the bacterial population was inactivated following five days of microcosm biofilm development on enamel surfaces [7]. On the other hand, there is evidence for a moderate antimicrobial effect (50–70%) in the case of *Hypericum perforatum* without activation, with a bacteriostatic rather than a bactericidal effect [14]. These effects may be related to hypericin—a well-known photosensitizer and sonosensitizer [18,47], whereas hyperforin accounts for intrinsic antibacterial activity, independent of photo- and sonoactivation [17]. Overall, our findings provide pertinent evidence that St. John’s wort extract may enhance the disruption and reduction in multispecies dental biofilms when applied alongside ultrasonic debridement under *in vitro* conditions. More precisely, this approach significantly decreases biofilm biomass, total viable bacterial counts, and *Porphyromonas gingivalis* genomic load while substantially perturbing biofilm architecture, with effects comparable to those obtained with chlorhexidine.

Consistent with other pilot investigations, several limitations should be considered when interpreting the results of the present study. First, this simplified, static, seven-day *in vitro* biofilm model on bovine dentin may not fully replicate *in vivo* oral conditions (e.g., salivary flow, host immune response, nutritional factors) and the anatomical complexity of a periodontal pocket. With multispecies communities and controlled *in vitro* conditions, however, this framework provides a more realistic perspective than monospecific biofilms. Despite the absence of host immune and inflammatory responses, this approach is also compatible with standard initial screening methodologies for novel antimicrobial agents, allowing the intrinsic antibacterial activity to be assessed without confounding host-related variables. Second, we did not determine the retention time and *in vivo* bioavailability of the hydroalcoholic extract of *Hypericum* perforatum, while the 30 s irrigation time does not reflect the ‘wash-out’ effect of human crevicular fluid flow. Nevertheless, the antimicrobial effect observed after clinically relevant exposure periods indicates that the extract possesses intrinsic antibacterial potential. In addition, treatment duration was selected to simulate the typical clinical contact time of ultrasonic instrumentation applied to a localized root surface during non-surgical periodontal therapy, rather than to mimic continuous fluid dynamics within the periodontal pocket. Third, the absence of cytotoxicity/tissue compatibility testing and unknown effects on recolonization or microbial resistance can be viewed as an additional drawback. In this context, it is worth mentioning that this study focused specifically on antimicrobial efficacy as an initial proof-of-concept. Biocompatibility and cytotoxicity assessments are essential subsequent steps, being planned for future investigations to determine the safety profile and therapeutic applicability of the extract. Furthermore, St. John’s wort contains multiple bioactive constituents that may decrease the likelihood of rapid resistance versus single-target antimicrobials [16,17,18,19,20,21]. Fourth, it should be noted that bacterial suspensions were combined based on equal optical density to standardize the initial inoculum and ensure experimental reproducibility. Although this approach does not fully reproduce the ecological composition and relative abundance of microorganisms present in natural periodontal biofilms, it is commonly used in *in vitro* polymicrobial models designed to evaluate antimicrobial effects under controlled conditions. Fifth, one can argue that ultrasonic energy may contribute to sonoactivation of hypericin since this compound exhibits photo- and potentially sonosensitizing properties. The ultrasonic instrumentation used here was applied under standard clinical debridement settings, being intended mainly for mechanical biofilm disruption and irrigation rather than for targeted sonodynamic activation. Although the possibility of partial ultrasonic activation of photosensitive compounds cannot be completely excluded, the present study was not designed to investigate this mechanism. Sixth, the relatively small sample size, the possibility of selection bias, and the risk of measurement bias may also represent limitations of the study. However, the present study was designed as a pilot exploratory investigation conducted under controlled experimental conditions to generate preliminary data. Finally, we did not conduct preliminary pilot testing or dose–response analysis in the present study, and only a single extract concentration was evaluated. Such an approach, involving the evaluation of a single working concentration, is commonly used in exploratory in vitro studies to establish preliminary evidence of antimicrobial activity before more detailed dose–response investigations are undertaken. Overall, our findings should be viewed as a proof-of-concept demonstrating the susceptibility of a multispecies biofilm to this extract, rather than a direct prediction of clinical success. Within this framework, longitudinal and repeated-exposure investigations are required to confirm these in vitro effects in relevant animal models and well-designed randomized controlled clinical trials. Future investigations could incorporate semi-quantitative approaches, such as live/dead fluorescence ratio analysis, to allow a more detailed assessment of treatment-induced changes in biofilm viability. Moreover, such studies should be designed to allow the assessment of *Hypericum perforatum* extract efficacy in reducing clinical parameters of periodontal disease (e.g., probing depth, clinical attachment level, bleeding on probing), its impact on the subgingival microbiome, and its safety and patient acceptance.

## 5. Conclusions

In this multispecies dentin-disc biofilm model, irrigation with a hydroalcoholic extract of *Hypericum perforatum* during ultrasonic debridement improved biofilm control compared with mechanical debridement alone or saline irrigation. The treatment was associated with reductions in biofilm biomass, viable bacterial counts, and *Porphyromonas gingivalis* genomic load, with antimicrobial effects comparable to chlorhexidine under the experimental conditions used. Confocal microscopy further indicated disruption of biofilm architecture and decreased bacterial viability. These data should be interpreted as proof-of-concept laboratory evidence demonstrating the antibiofilm potential of *H. perforatum* extract under controlled *in vitro* conditions. Whether these effects translate into clinically meaningful improvements in periodontal outcomes remains uncertain and requires further evaluation in preclinical models and well-designed clinical studies.

## Data Availability

All the data generated or analyzed in this study are included in this published article.

## Supplementary Materials

The following supporting information can be downloaded at https://www.mdpi.com/article/10.3390/medicina62030563/s1. Table S1: Biofilm biomass (optical density at 595 nm) after treatments; Table S2: Mean total viable bacterial count (log10 CFU/disc) after treatments; Table S3: qPCR data (log10 DNA copies/disc) for *Porphyromonas gingivalis*.

## Abbreviations

The following abbreviations are used in this manuscript:

SC	Saline Control
UD	Ultrasonic Debridement
CHX	Chlorhexidine
SJW	St. John’s wort (*Hypericum perforatum*)
CLSM	Confocal Laser Scanning Microscopy
CFU	Colony-Forming Units
qPCR	Quantitative Polymerase Chain Reaction
BHI	Brain Heart Infusion
ATCC	American Type Culture Collection
OD	Optical Density
ANOVA	Analysis of Variance
DMSO	Dimethyl Sulfoxide
iNOS	Inducible Nitric Oxide Synthase
NF-κB	Nuclear Factor Kappa B
DNA	Deoxyribonucleic Acid
RNA	Ribonucleic Acid
OD600	Optical Density at 600 nm
