# Supplementary material for: In Vitro Efficacy of Ultrasonic Debridement with Adjunctive St. John’s Wort on Multispecies Dental Biofilms
Source: Medicina (Kaunas). 2026 Mar 18;62(3):563. doi: 10.3390/medicina62030563 (PMC13027743; doi:10.3390/medicina62030563)
Supplement: Supplementary file 1 [file medicina-62-00563-s001.zip › medicina-4163305-supplementary.pdf]

**Table S1.** Biofilm biomass (optical density at 595nm) after treatments.

| Treatment group | Mean OD | % Reduction from untreated control | Statistical significance (vs. untreated control) | Statistical significance (vs. UD Alone) |
|---|---|---|---|---|
| UC | 1.25 ± 0.15 | - | - | - |
| SC | 1.18 ± 0.12 | 5.6% | 0.45 | - |
| UD | 0.55 ± 0.08 | 56.0% | **< 0.001 ***** | - |
| UD + CHX | 0.30 ± 0.05 | 76.0% | **< 0.001 ***** | **0.001 **** |
| UD + SJW | 0.35 ± 0.06 | 72.0% | **< 0.001 ***** | **0.003 **** |

UC, untreated control; SC, saline control; UD, ultrasonic debriment alone; UD + CHX, ultrasonic debriment with chlorhexidine; UD + SJW, ultrasonic debriment with St. John's wort. Data in the second column represent mean optical density values with one standard deviation. Data in the fourth and fifth columns indicate *p*-values obtained from post hoc comparisons against the untreated control and against ultrasonic debridement alone, respectively. Bold values annotated with asterisks (*) denote significant differences (Tukey's HSD test, ***—$p \leq 0.001$, **—$p \leq 0.01$, and *—$p \leq 0.05$).

**Table S2.** Mean total viable bacterial count (log10 CFU/disc) after treatments

| Treatment group | Mean $Log_{10}$ CFU/disc | % Reduction from untreated control | Statistical significance (vs. untreated control) | Statistical significance (vs. UD Alone) |
|---|---|---|---|---|
| UC | 8.5 ± 0.3 | - | - | - |
| SC | 8.3 ± 0.3 | 2.4% | 0.21 | - |
| UD | 6.2 ± 0.4 | 27.05% | **< 0.001 ***** | - |
| UD + CHX | 4.5 ± 0.4 | 47.05% | **< 0.001 ***** | **0.002 **** |
| UD + SJW | 4.8 ± 0.5 | 43.52% | **< 0.001 ***** | **0.005 **** |

UC, untreated control; SC, saline control; UD, ultrasonic debriment alone; UD + CHX, ultrasonic debriment with chlorhexidine; UD + SJW, ultrasonic debriment with St. John's wort. Data in the second column represent mean optical density values with one standard deviation. Data in the fourth and fifth columns indicate *p*-values obtained from post hoc comparisons against the untreated control and against ultrasonic debridement alone, respectively. Bold values annotated with asterisks (*) denote significant differences (Tukey's HSD test, ***—$p \leq 0.001$, **—$p \leq 0.01$, and *—$p \leq 0.05$).

**Table S3.** qPCR data (log10 DNA copies/disc) for *Porphyromonas gingivalis*

| Treatment group | Mean Log10 DNA copies/disc | % Reduction from untreated control | Statistical significance (vs. untreated control) | Statistical significance (vs. UD Alone) |
|---|---|---|---|---|
| UC | 6.8 ± 0.2 | - | | - |
| SC | 6.0 ± 0.3 | 11.76% | | - |
| UD | 4.5 ± 0.3 | 33.82% | **< 0.001** *** | - |
| UD + CHX | 2.6 ± 0.3 | 61.76% | **< 0.001** *** | **0.006** ** |
| UD + SJW | 3.0 ± 0.2 | 55.88% | **< 0.001** *** | **0.009** ** |

UC, untreated control; SC, saline control; UD, ultrasonic debriment alone; UD + CHX, ultrasonic debriment with chlorhexidine; UD + SJW, ultrasonic debriment with St. John's wort. Data in the second column represent mean optical density values with one standard deviation. Data in the fourth and fifth columns indicate $p$-values obtained from post hoc comparisons against the untreated control and against ultrasonic debridement alone, respectively. Bold values annotated with asterisks (*) denote significant differences (Tukey's HSD test, ***—$p \leq 0.001$, **—$p \leq 0.01$, and *—$p \leq 0.05$).
